# Effects of Total Resources, Resource Ratios, and Species Richness on Algal Productivity and Evenness at Both Metacommunity and Local Scales

**DOI:** 10.1371/journal.pone.0021972

**Published:** 2011-07-06

**Authors:** Lars Gamfeldt, Helmut Hillebrand

**Affiliations:** 1 Institute for Botany, University of Cologne, Cologne, Germany; 2 Department of Marine Ecology, University of Gothenburg, Gothenburg, Sweden; 3 Institute for Chemistry and Biology of the Marine Environment (ICBM), Carl-von-Ossietzky University Oldenburg, Oldenburg, Germany; University of Liverpool, United Kingdom

## Abstract

The study of the interrelationship between productivity and biodiversity is a major research field in ecology. Theory predicts that if essential resources are heterogeneously distributed across a metacommunity, single species may dominate productivity in individual metacommunity patches, but a mixture of species will maximize productivity across the whole metacommunity. It also predicts that a balanced supply of resources within local patches should favor species coexistence, whereas resource imbalance would favor the dominance of one species. We performed an experiment with five freshwater algal species to study the effects of total supply of resources, their ratios, and species richness on biovolume production and evenness at the scale of both local patches and metacommunities. Generally, algal biovolume increased, whereas algal resource use efficiency (RUE) and evenness decreased with increasing total supply of resources in mixed communities containing all five species. In contrast to predictions for biovolume production, the species mixtures did not outperform all monocultures at the scale of metacommunities. In other words, we observed no general transgressive overyielding. However, RUE was always higher in mixtures than predicted from monocultures, and analyses indicate that resource partitioning or facilitation in mixtures resulted in higher-than-expected productivity at high resource supply. Contrasting our predictions for the local scale, balanced supply of resources did not generally favor higher local evenness, however lowest evenness was confined to patches with the most imbalanced supply. Thus, our study provides mixed support for recent theoretical advancements to understand biodiversity-productivity relationships.

## Introduction

The relationship between productivity and biodiversity of ecosystems has been, and continues to be, a major focus of ecological research [1]–[3]. Synthetic work has shown that the shape of this relationship is highly variable, from no obvious relationship, to hump-shaped, negative, and positive [2], [4]. There have been numerous attempts to explain why diversity could be expected to change with changes in productivity [5]–[7]. However, progress in our understanding of the mechanisms behind observed productivity-diversity relationships has been hampered by a lack of clear definitions of productivity. Estimates of productivity range from the direct measure of the rate of carbon flux through organisms, to standing stock biomass, to rates of resource supply, to derived variables such as latitude and precipitation (see [3] for an overview). It has often been implicitly assumed that these estimates are interchangeable [5], whereas in fact they are most often not [2]. Another issue that limits our understanding of the productivity-diversity relationship is the direction of causality. While ecologists have traditionally viewed biodiversity as a function of productivity [2], [5], [8], recent work has explored the relationship from a fundamentally differing perspective, where productivity is viewed as a function of changes in biodiversity [9]. This has spurred a lively debate about whether biodiversity is the cause or consequence of the productivity of ecosystems (see e.g. [10], [11]). A rich body of research that manipulated the richness of species has found strikingly general patterns in the way biodiversity affect resource capture and productivity [12]. The mean effect of increasing richness in these studies is to increase both biomass production and depletion of resources [13], [14].

Recent theoretical and empirical advancements may aid in a consolidation of our view of the productivity-biodiversity relationship. A first step is the realization that both productivity and biodiversity respond to the magnitude and the balance of resource supply [3], [11], [15]. The latter argument on the stoichiometry of resources is well known from competition theory in the form of resource-ratio theory [16], but has rarely been explicitly addressed in assessments of productivity and diversity. In a mathematical model with metacommunities, Gross and Cardinale [11] show that both the supply rate and ratio of limiting resources determined realized richness and productivity. The model emphasizes three major points. The first one is that the often-suggested hump-shaped response of diversity to changes in productivity is due to the fact that species are able to coexist via niche partitioning only at intermediate levels of resource ratios. This mechanism, by which resource availability affects diversity, is fundamentally connected to the way diversity affects resource use and biomass production. When resource supply does not permit species coexistence, resource use and biomass are driven by the competitively dominant species (as observed in many classical ecological experiments, e.g. [17], [18]). When resource levels allow coexistence, resource partitioning results in transgressive overyielding [19], with more diverse assemblages experiencing more effective resource use than any single species. It has indeed been shown experimentally that a diverse community (of bacteria) in which species differ slightly in their efficiency in exploiting different resources is more fully using the available niche space, thereby enhancing ecosystem processes [20]. The second point in the model is that resource supply can affect diversity and mediate the way diversity affects resource use at the same spatial scale. Third, even though total biomass of species and their diversity may often be correlated, one should caution to make inference about direct mechanistic relationships based on the observation of the two alone. Biomass and diversity are affected by variations in resource supply, and changes in resources are likely to affect actual levels of both as well as their interrelationship. It should be noted that there of course are other factors that are important in affecting species coexistence, such as dispersal, disturbances, and pathogens. The value of focusing on multiple limiting resources should foremost be evident in systems where resource competition is strong.

For a mechanistic understanding of the relationship between resources and diversity it is thus important to distinguish between the total rate of supply of resources and their ratios (resource stoichiometry). Resources are often patchily distributed in natural systems [21]–[23] and are required at different amounts and ratios by different species [24]. Species often have trade-offs in their resource use efficiency [16], [17] and theory predicts that species cannot simultaneously be the best competitor for all resources. At extreme resource ratios most species are likely to be limited by the same resources with little chance for competitive coexistence. Multiple limiting resources that are, on average, in a balanced supply should allow for more species to coexist than should resources with a highly skewed distribution [25]. Since resource ratios influence species coexistence, they indirectly also affect community biomass and productivity. In an experiment with microalgae, Hillebrand and Lehmpfuhl [26] manipulated total resource supply and resource ratios in a metacommunity framework. Each metacommunity consisted of three patches differing in their resource ratios. Results showed that total resource supply increase biomass but decrease species richness and evenness. In addition, algal resource use efficiency increase with increasing richness and evenness at the scale of whole metacommunities indicating that resource stoichiometry is an important mediator of the relationship between diversity and resource uptake. However, since the experiment did not directly manipulate species richness it could not evaluate whether a mix of species has higher resource use efficiency than single species at the scale of patches (each with a unique resource ratio) or metacommunities (consisting of three patches with unique resource ratios).

To explicitly study how algal species richness, resource supply and resource ratios interact to influence resource use efficiency, biovolume and diversity (expressed as algal evenness), we manipulated these three factors in experimentally assembled freshwater metacommunities. At the metacommunity (i.e. regional) scale we manipulated the amount of total phosphorus. Since the ratio of limiting resources can affect species coexistence, and thereby also resource use efficiency, we used metacommunities consisting of three local patches, where patches differed in the ratio of nitrogen and phosphorus. Whereas one or a few species can be expected to competitively exclude other species in local patches, more species can potentially coexist at the metacommunity scale when there is spatial turnover in resource ratios and species differ in their requirements for limiting resources. If resource use efficiency does in fact differ among species under different resource ratios, it could be hypothesized that a mixture of species enhances resource use efficiency at the metacommunity scale compared to single species. This hypothesis was not tested in the study by Hillebrand and Lehmpfuhl [26]. We compared species responses in mixtures to expectations derived from the component algal monocultures to study how species richness affects resource use efficiency and algal biovolume. Specifically, we tested the following hypotheses on the scale of both local patches and metacommunities:

H1: Increasing total amount of resources increases algal biovolume at both local patch and metacommunity scales.

H2: Imbalance in resource supply decreases realized algal diversity (evenness) in species mixtures treatments at the local scale.

H3: Resource use efficiency and productivity increase with algal richness at the metacommunity scale since the species with the highest resource use efficiency differs among patches with different resource ratios. Metacommunities consisting of only single species will thus have high biovolume in some local patches but not in others, whereas metacommunities consisting of all species will have high biovolume in all local patches. At the local scale, however, richness will have no effect since single species maximize biovolume production.

H4: Realized algal evenness is positively correlated with resource use efficiency, and thus also realized algal biovolume, at the metacommunity scale. This pattern was observed in the previous study using a similar experimental setup as this experiment [26].

## Methods

### Species

We used five species of freshwater microalgae: *Ankistrodesmus* sp. (Chlorophyta), *Chlamydomonas terricola* (Chlorophyta), *Cylindrospermum* sp. (Cyanobacteria), *Fragilaria sapucina* (Bacillariophyta), and *Gymnodinium* sp. (Dinophyta). All species are hereafter referred to by their genus names only. Strains were obtained from the CCAC culture collection of algae in Cologne, Germany, and were kept in WC medium [27] in pre-cultivation and during the experiment. We chose these taxa to represent a wide range of resource requirements: chlorophytes have high requirements for nitrogen, *Cylindrospermum* can fix inorganic nitrogen, diatoms need large amounts of silicate, and dinoflagellates have high requirements for carbon (e.g. [28]).

### Experimental design

We simultaneously manipulated species composition, total supply of phosphorus (P_sup_), and nitrogen to phosphorus (N∶P) ratios in a factorial experiment. Species composition had six levels: each species in monoculture and a mixture of all five species. We used a substitutive design with a constant starting biovolume of 231*10^3^ µm^3^ mL^−1^ for both monocultures and mixture, and the total biovolume in the mixture evenly divided among the five species. There were three levels of total amount of phosphorus to reflect the range of phosphorus loading encountered in natural freshwater systems [29]: 0.13, 0.81, and 5.02 µmol L−1. We created metacommunities (replicated three times within each level of total phosphorus) consisting of three local 50 ml patches (in 60 ml Nunc flasks), with N∶P ratios of 2, 16, and 128 respectively. Our choice of 16 as an intermediate level was based on the Redfield ratio [30]. Since P_sup_ was fully crossed with N∶P, it follows that increasing P_sup_ was associated with increasing nitrogen. Dispersal between local patches within each metacommunity was carried out three times during the experiment (days 13, 20 and 27) by removing 2.5 ml from each local patch, thoroughly mixing the 7.5 ml, and then redistributing 2.5 ml to each patch.

For additional information on how species richness and resource supply affected algal biovolume and resource use efficiency we included two extra levels of total phosphorus: 0.32 and 2.02 µmol L-1. These levels were crossed with the species mixture treatment only, yielding 18 extra flasks (grand total number of flasks = 180). Throughout the paper, the five levels of total phosphorus (0.13, 0.32, 0.81, 2.02, and 5.02) are referred to by the roman numbers I, II, III, IV, and V respectively.

The experiment ran for 31 days between February 21^st^ and March 23^rd^ 2007 at the Institute for Botany, University of Cologne (latitude 50° 55′ 48″, longitude 6° 55′ 12″). Flasks were placed in a climate chamber set to 15°C with a 12 h: 12 h light: dark cycle, with each flask assigned a random position in the chamber. We exchanged medium on days 3, 6, 10, and 17 of the experiment by pipetting 5 mL (day 3 and 6) or 10 mL (the other dates) and replacing the removed volume with fresh medium. In total, we removed and replaced 10 ml from each local patch every week. The lids on the flasks had filtered ventilation holes to allow gas exchange while at the same time preventing cross-contamination. Each flask was gently stirred every second day to keep cells in suspension. At the same time, flasks were randomly redistributed to a new position to avoid effects of small-scale variation in light intensity in the climate chamber. All culturing and experimental work was performed under sterile conditions on a clean-bench, and all material was autoclaved and HCl-washed.

### Dependent variables

At the end of the experiment we took samples for nutrient analyses. Samples were analyzed for nitrogen and phosphorus using an autoanalyser. For each local patch we determined population sizes by counting samples in Utermöhl chambers under a Leica DMIRB inverted microscope. Total algal biovolumes were determined by multiplying the number of cells by their biovolume. Mean biovolumes were based on the geometric shape of each species as described in [31], and estimated by measuring the dimensions of 25 cells per species before the experiment. Depending on species type and cell densities, samples were sometimes diluted and counted under 100, 200 or 400 times magnification using a counting grid. For monocultures, at least 400 cells and ten grids were counted, and for mixtures a minimum of 1000 cells and ten grids. Biovolume was our estimate of realized production and biovolume proportions of species were used to calculate Pielou's Evenness index [32]. We analyzed the effect of both resource supply and ratio on evenness at the local scale, and resource supply alone on evenness at the metacommunity scale. Metacommunity evenness was calculated based on the total biovolume of all algal species across all three local patches. We calculated a measure of resource use efficiency (RUE), which was final algal biovolume per initial unit phosphorus (P_sup_). At the metacommunity scale, RUE was calculated as the mean algal biovolume across local patches divided by P_sup_. All data on biovolume, resources, and resource use efficiency were ln-transformed prior to statistical analyses. To explore the effect of species richness on biovolume, as compared to monocultures, we calculated net diversity effects (*sensu* Loreau and Hector [33]) and divided these into selection effects (the effects of individual species) and complementarity (e.g. effects of niche partitioning and facilitation). We also compared patterns of RUE (Δ_RUE_) and evenness (Δ_Even_) in the algal mixture to the patterns expected based on species performances in monocultures. Δ_RUE_ could not be partitioned into complementarity and selection because species-specific contributions to RUE in the mixture could not be quantified.

### Statistical analyses

For analyses at the local scale on the effects of resource supply on algal biovolume and evenness we used a two-factor ANOVA (with α = 0.05) with total phosphorus (P_sup_) and N∶P ratio as factors. When testing for the effects of initial species composition and richness we used a three-factor ANOVA with species composition, P_sup_ and N∶P ratio as factors. At the metacommunity scale, data were also analyzed with ANOVA with the exception that N∶P ratio was not included (since there was no manipulation of N∶P at this scale).

Patches within metacommunities in our experiment were not strictly independent, as 5% of patch volume was exchanged each week. This introduces a source of interdependence in the data, which makes patches of different N∶P within metacommunities slightly more similar compared to patches among metacommunities. Such interdependence represents an inherent problem in the analysis of metacommunity studies. Previous studies have either focused on independently replicated regional patterns only or used bootstraps with the true number of replicates to assess local patterns [34]. Focusing on regional metacommunity patterns only would force us to ignore the results of important interactions at the local scale. This would be unfortunate since we were directly interested in local interactions between species composition (species identity and richness) and resource ratios. A bootstrapping approach would collapse our multi-scale design, allowing us to only explore effects at the metacommunity scale. For the purposes of our analyses, we argue that the interdependence among local patches in our experiment blurs rather than strengthens differences among treatments of different N∶P ratios: dispersal was only among patches of different N∶P, not among metacommunities. Moreover, we approached the test of hypotheses on the local scale with additional conservatism in terms of the degrees of freedom (see also [26]): for each of the response variables at the local scale, we conducted factorial ANOVAs in which the F-ratios retrieved were tested for significance at α = 0.05 using an F-distribution with a third (because there are 3 dependent patches in each metacommunity) of the actual degrees of freedom in the error term. Significant local treatment effects were those that showed an observed F_(x;df)_ larger than the critical F_(x;df)_.

To explore hypothesis 4 we did correlations between observed patterns of realized algal evenness and RUE, at both local and metacommunity scales. We also did the correlations between realized algal evenness and RUE for the treatment residuals to evaluate if the relationship changes. If the results from the correlations on observed patterns and residuals differ, observed patterns may be largely due to treatment effects.

## Results

### Effects of P_sup_ and N∶P

**Figure 1:**
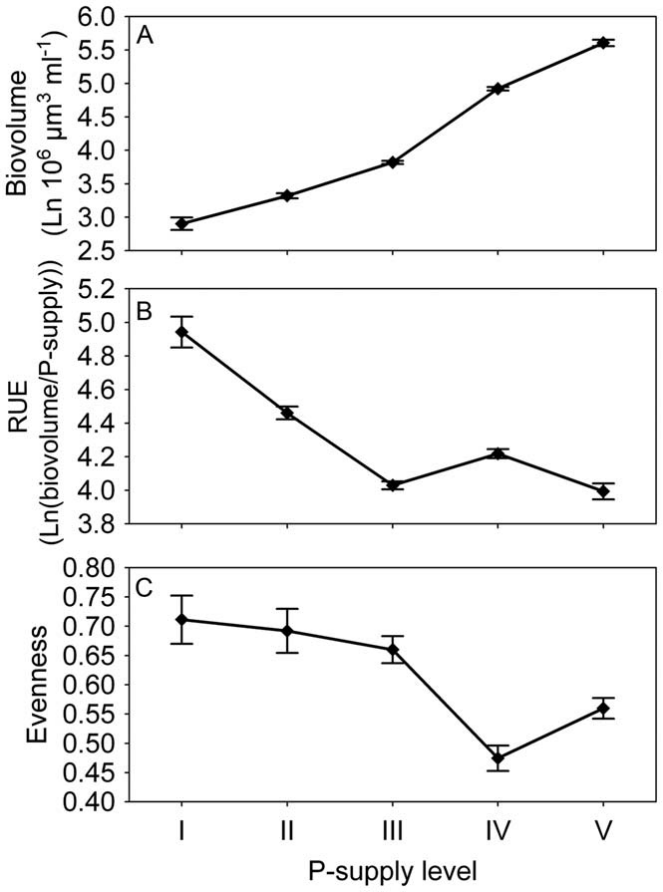
Effects of phosphorus supply (P_sup_) at the metacommunity scale. P_sup_ generally increased algal biovolume (A), decreased resource use efficiency (B), and decreased evenness (C). Results are based on the mixture treatments only. For this and the following figures, all error bars are 1 standard error.

**Figure 2:**
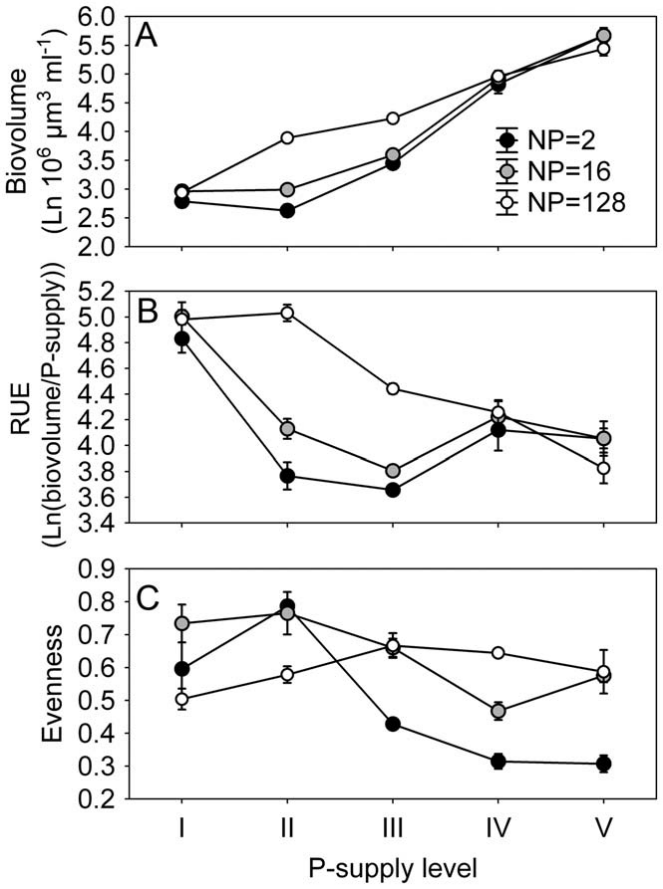
Effects of phosphorus supply (P_sup_) and nitrogen∶phosphorus (N∶P) ratio at the local scale. Algal biovolume generally increased (A) and resource use efficiency decreased (B) with increasing P_sup_. There were mixed effects of P_sup_ and N∶P on evenness (C). Results are based on the mixture treatments only.

At the metacommunity scale, biovolume increased and resource use efficiency (RUE) decreased with increasing P_sup_ (Table 1A; Figs. 1A, B respectively). Evenness was high at low levels, and low at high levels of P_sup_ (Fig. 1C). The general patterns on the local scale matched those on the metacommunity scale (Fig. 2), and there were significant and interactive effects of P_sup_ and N∶P ratio on algal biovolume (Table 1B). Algal biovolume increased by a factor ranging from around 56 (at P_sup_ I) to around 1500 (at P_sup_ V) over the course of the experiment compared to inoculated biovolumes. At the local scale, algal biovolume generally increased with P_sup_ and there were no differences among N∶P ratios at low and high levels of P_sup_ (Fig. 2A). At P_sup_ II and III, however, biovolume was highest for the highest N∶P ratio. RUE generally decreased with increasing P_sup_ and was consistently lowest for the treatments with highest biovolume, and vice versa (Fig 2B). Effects of P_sup_ and N∶P ratios on algal evenness were highly variable (Table 1B; Fig. 2C). With respect to P_sup_ there was a trend that evenness was hump-shaped at N∶P ratio 128, flat at N∶P ratio 16, and declining at N∶P ratio 2.

**Table 1:** Effects of total phosphorus (P_sup_) and nitrogen∶phosphorus ratio (N∶P) on algal biovolume, resource use efficiency (RUE), and algal evenness on (A) metacomunnity and (B) local scales.

A.		Biovolume		RUE		Evenness	
Factor	Df (adj)	F	P adj	F	P adj	F	P adj
P_sup_	4	470	**<0.001**	49.5	**<0.001**	11.3	**0.0010**
Error	10						

B.							
P_sup_	4	411	**<0.001**	48.7	**<0.001**	12.3	**<0.001**
N∶P	2	23.5	**<0.001**	23.51	**<0.001**	14.1	**0.0012**
P_sup_*N∶P	8	10.4	**<0.001**	10.4	**<0.001**	7.36	**0.0025**
Error	30 (10)						

Results as analyzed with ANOVA with adjusted (adj) number of degrees of freedom in the error term (see text for details). Significant results are shown in bold.

### Effects of species richness and identity

**Figure 3:**
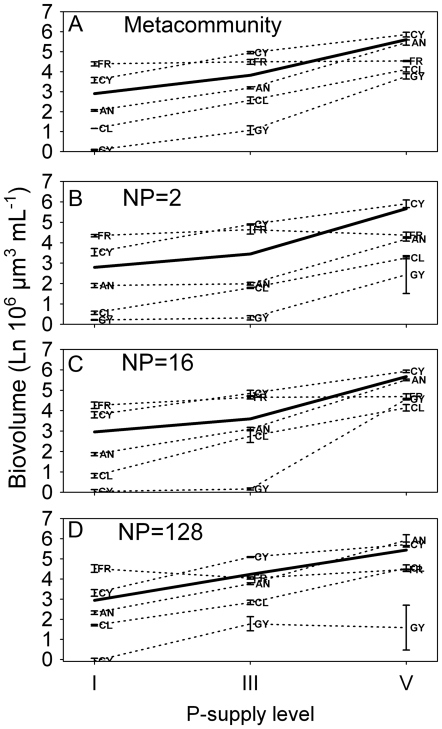
Algal composition and biovolume. Algal biovolume for the six different algal compositions, five monocultures and the mixture, under different levels of P_sup_. (A) The metacommunity scale, and (B–D) local patch scale for the three levels of N∶P. AN = *Ankistrodesmus*, CL = *Chlamydomonas*, CY = *Cylindrospermum*, FR = *Fragilaria*, and GY = *Gymnodinium*. Solid line indicates the mixture.

Whereas total algal biovolume at the metacommunity scale was as high in the mixture as in the best monocultures (*Cylindrospermum* and *Ankistrodesmus*) at the highest P_sup_ level, it was not at the lower levels (Table 2A; Fig. 3A). *Fragilaria* had highest biovolume at P_sup_ I and *Cylindrospermum* at P_sup_ III. Information from the local patch scale showed that the green alga *Ankistrodesmus* had as high biovolume as *Cylindrospermum* when supply of N was high (at N∶P ratio 128; Fig. 3D). RUE matched the results on biovolume well, i.e. species with low biovolume had low RUE and vice versa (Fig. S1).

**Table 2:** Effects of P_sup_, N∶P, and Sp (species composition: individual species and mixture) on algal biovolume and RUE on (A) metacommunity and (B) local scales.

A.		Biovolume		RUE	
Factor	Df (adj)	F	P adj	F	P adj
P_sup_	2	842	**<0.001**	177	**<0.001**
Sp	5	376	**<0.001**	376	**<0.001**
P_sup_*Sp	10	39.7	**<0.001**	39.7	**<0.001**
Error	36				

B.					
P_sup_	2	458	**<0.001**	149	**<0.001**
N∶P	2	19	**<0.001**	19.0	**<0.001**
Sp	5	296	**<0.001**	296	**<0.001**
P_sup_*N∶P	4	7.96	**<0.001**	7.96	**<0.001**
P_sup_*Sp	10	20.0	**<0.001**	20.0	**<0.001**
N∶P*Sp	10	6.11	**<0.001**	6.11	**<0.001**
P_sup_*N∶P*Sp	20	5.19	**<0.001**	5.19	**<0.001**
Error	108 (36)				

Details as in Table 1.

**Figure 4:**
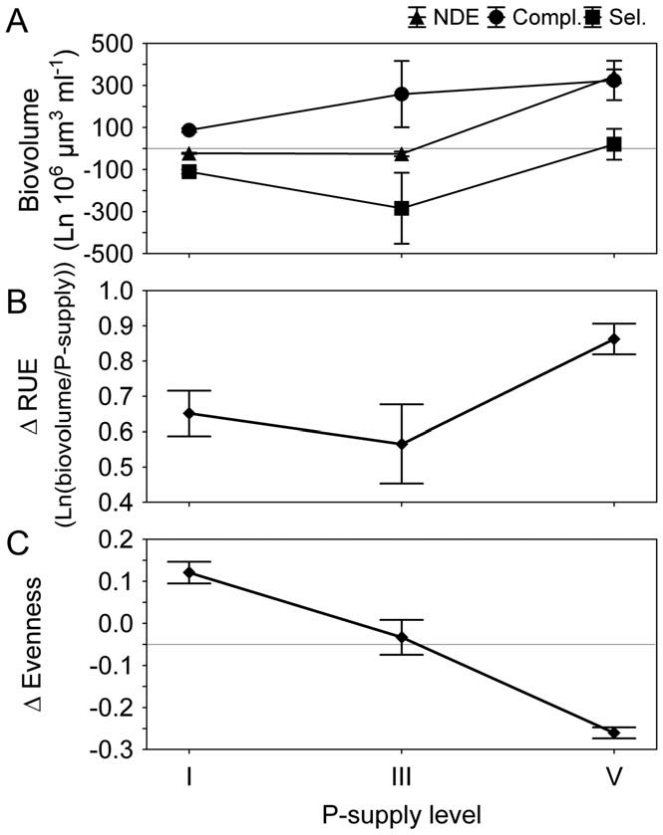
Comparing observed and expected results for the mixture (diversity effects) at metacommunity scale. Net diversity, complementarity, and selection effects for algal biovolume (A). Net diversity effects for resource use efficiency, Δ_RUE_ (B). The difference between evenness (Δ_Evenness_) in mixture and in monocultures (C).

Net diversity effect for biovolume production at the metacommunity scale was positive (i.e. significantly larger than zero) only at P_sup_ V, whereas complementarity effect was positive at P_sup_ I and selection effects negative or not different from zero irrespective of P_sup_ (Table 3A; Fig. 4A). Positive complementarity effects suggest that algal species showed niche partitioning or positive interactions whereas absence of positive selection effects inferred that those species that were most productive in monocultures did not dominate the species mixture. Net diversity effects for RUE (Δ_RUE_) were positive throughout the experiment, thus mixtures always used P more efficiently than predicted from the monocultures (Table 3A; Fig. 4B). For evenness (Δ_Even_), mixtures were more even at P_sup_ I compared to what would be expected based on how species grew in the absence of interspecific competition (monocultures), whereas the opposite was true at P_sup_ V, when mixtures were less even than expected (Table 3A; Fig. 4C).

**Table 3:** Results of t tests to analyze if net diversity effects, complementarity effects, selection effects, Δ_RUE_, and Δ_Even_ are different from zero on (A) metacomunnity and (B) local scales.

A.	Net div.		Compl.		Sel.		Δ_RUE_		Δ_Even_	
Factor	t	P	t	P	t	P	t	P	t	P
P_sup_ I	−8.0	**0.015**	9.4	**0.011**	−10.6	**0.0087**	10.1	**0.0096**	4.7	**0.042**
P_sup_ III	−2.2	0.16	1.6	0.24	−1.7	0.23	5.0	**0.037**	−0.80	0.51
P_sup_ V	10.6	**0.0088**	3.5	0.075	0.28	0.81	19.8	**0.0025**	−20.3	**0.0025**

B.										
P_sup_ I, 2	−6.1	**0.026**	4.2	0.052	−4.6	**0.044**	8.3	**0.014**	0.23	0.84
P_sup_ I, 16	−3.2	0.085	30.3	**0.0011**	−16.9	**0.0035**	25.8	**0.0015**	3.2	0.086
P_sup_ I, 128	−2.9	0.10	0.82	0.50	−1.7	0.24	4.0	0.058	−1.6	0.25
P_sup_ III, 2	−3.2	0.081	1.6	0.26	−1.9	0.20	13.2	**0.0057**	−20.8	**0.0023**
P_sup_ III, 16	−4.2	0.052	1.2	0.34	−2.0	0.18	6.6	**0.022**	−2.2	0.16
P_sup_ III, 128	3.7	0.065	1.5	0.27	−1.2	0.34	6.3	**0.024**	−1.4	0.31
P_sup_ V, 2	8.3	**0.014**	9.4	**0.011**	4.0	0.058	14.8	**0.0045**	−14.3	**0.0048**
P_sup_ V, 16	5.2	**0.035**	12.6	**0.0062**	−0.86	0.48	24.0	**0.0017**	−7.3	**0.018**
P_sup_ V, 128	1.6	0.25	1.0	0.41	−0.56	0.63	7.0	**0.020**	−3.1	0.089

3 levels of total phosphorus (P_sup_) and 3 levels of N∶P ratio (shown after comma sign as 2, 16 or 128). Δ_RUE_ and Δ_Even_ are observed resource use and observed evenness compared to expected values in monocultures. Significant results are shown in bold.

Proportional species biovolume in the mixtures qualitatively resembled those observed for the monocultures, but the absolute deviation differed (Fig. S2). For example, observed proportions of the algal species in mixtures showed that the dominance of *Cylindrospermum* increased as phosphorus became less limiting, and *Fragilaria* and *Cylindrospermum* were equally abundant at low P_sup_ (Fig. S2 A). Looking at the proportional biovolume of each species in monoculture compared to the total biovolume of all species monocultures (as an estimation of expected proportions in the mixture; see Fig. S2 for an explanation of calculations), *Cylindrospermum* was not as abundant as *Fragilaria* at low P_sup_ and not as dominant at high P_sup_ (Fig. S2 B). In terms of absolute values of biovolume, all species but *Gymnodinium* had higher biovolume than expected at highest P_sup_ level (Fig. S2 C).

**Figure 5:**
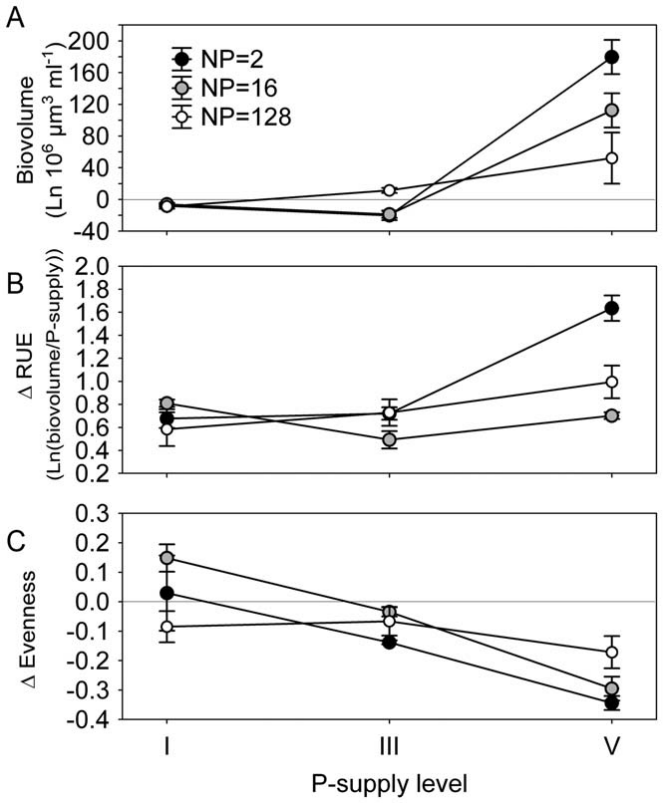
Comparing observed and expected results for the mixture (diversity effects) at local scale. Net diversity effects for algal biovolume (A). Net diversity effects for resource use efficiency, Δ_RUE_ (B). The difference between evenness (Δ_Evenness_) in mixture and in monocultures (C).

At the local patch scale, net diversity effects for biovolume were positive at P_sup_ V, where an increase with decreasing N∶P ratio was obvious (Table 3B; Fig. 5A). Complementarity effects were generally positive and selection effects generally negative, even though often not significantly so (Table 3B). As for the regional scale, local Δ_RUE_ was significantly positive throughout, and at high P-supply, Δ_RUE_ was higher for imbalanced than for balanced N∶P ratios (Fig. 5B). Evenness was lower than expected for intermediate and low N∶P, at P_sup_ V (Table 3C; Fig 5C).

### Correlation between evenness and other variables

**Figure 6:**
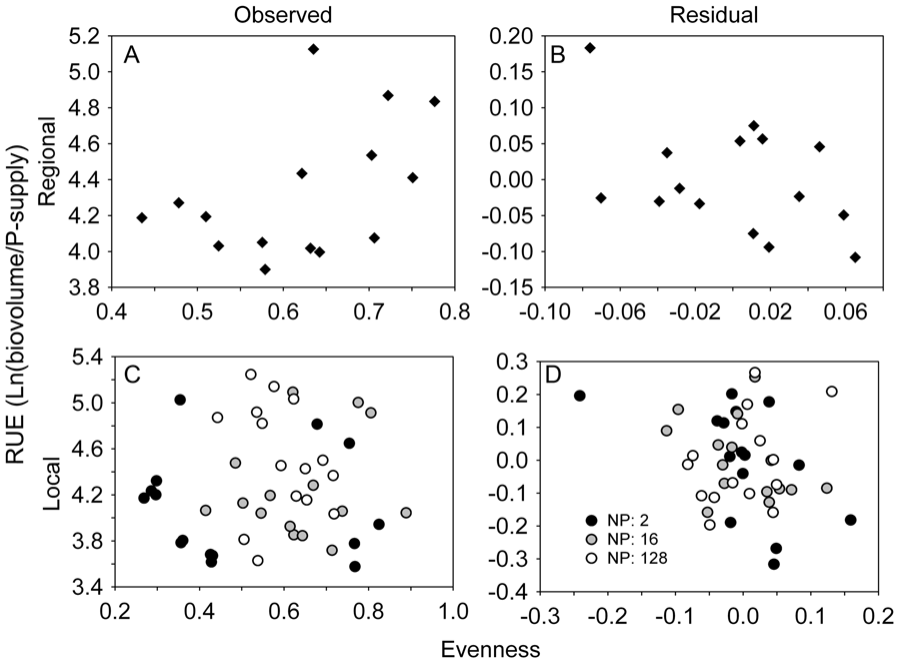
Correlation of algal evenness and resource use efficiency. At the regional (A,B) and local (C,D) scale. A and C are raw observed data, B and D residuals after analyzing the response of both resource use efficiency and evenness to the resource supply treatments.

We observed a marginally non-significant increase in RUE with increasing evenness at the regional scale (r = 0.47, p = 0.076) (Fig. 6A), but this correlation reversed when analyzing the treatment residuals for RUE and evenness instead (r = −0.447, p = 0.096, Fig. 6B). Thus, we cannot exclude that the positive correlation between RUE and evenness stems from their response to treatments alone. At the local scale, no significant correlations were found except for a marginally non-significant negative trend in the analysis on residuals (Fig. 6 C, D).

## Discussion

Recent studies have made significant progress in our understanding of the reciprocal effects of biodiversity and productivity through theory, field observations and experiments. In this experiment we explicitly studied how resource supply, resource ratios, and species richness interacted to affect resource use efficiency, producer biovolume, and evenness. There were clear effects at both metacommunity and local scales of increasing total resource supply (P_sup_) on algal biovolume, thereby accepting hypothesis 1. This is consistent with previous work that manipulated the supply of nitrogen and phosphorus [35], and with the previous study that used a similar design as our study [26]. Resource use efficiency, on the other hand, decreased with increasing P_sup_. Thus, when resources were more abundant, the algal communities became less efficient in turning these resources into new tissue.

Observed evenness declined with increasing P_sup_ at the metacommunity scale. The deviance of observed from expected evenness also declined, being positive at low and negative at high P_sup_. This indicates that species interactions tended to decrease evenness at high P_sup_, but to increase evenness at low P_sup_. By looking at the observed proportional biovolume of species in the mixtures compared to what would be expected from monocultures (Fig. S2) we get some insight into why this is so. The most striking differences between observed and expected patterns were that *Fragilaria* was less common than expected in mixtures at lowest P_sup_ thereby increasing evenness, whereas *Cylindrospermum* was more common and *Ankistrodesmus* less common in mixtures at highest P_sup_ thereby decreasing evenness (compare Figs. S2 A and S2 B). *Cylindrospermum* became increasingly competitively superior at the metacommunity scale when resources were more abundant.

Theory predicts that, on a local scale under stable conditions, the most efficient species will eventually outcompete all other species when competing for a single limiting resource [16]. For species to coexist they should be limited by different resources (even though other factors such as microbe composition can also affect coexistence and resource use, e.g. [36]). For example, a study with two freshwater diatom species showed that only when each species was limited by a different resource did coexistence occur [17]. Consequently, we hypothesized that a balanced supply of N∶P would favor the coexistence of species with both high (e.g. chlorophytes) and low (e.g. cyanobacteria) nitrogen requirements, and that deviations from such balance would result in competitive exclusion (see [11]). However, contrary to hypothesis 2 we found no general increase in evenness with balanced resource supply (N∶P = 16), thereby corroborating the findings of Hillebrand and Lehmpfuhl [26]. We observed no large shifts in the rank order of biovolume of species under different resource ratios when in monoculture (Figs. 3B–D), indicating that in the absence of competition, the algae did not perceive the metacommunities as very patchy. There were, however, some clear deviations from these patterns. For example, at P_sup_ V *Ankistrodesmus* ranked as the third most abundant species at lowest N∶P ratio, but was ranked first at highest N∶P. It thus seems as if some species indeed differed in their response to different resource ratios, and that this response depended on the total magnitude of resources available. We found an imprint of stoichiometry on dominance in that evenness was lowest in the combination of low P-supply and very high N∶P and high P-supply with very low N∶P, i.e. the most imbalanced actual supply rates in our experiment (Fig. 2). The patchiness as perceived by the algae, however, was not large enough to result in distinct community compositions under different resource ratios. A possible explanation for a lack of clear relationships between N∶P and evenness may be that dispersal of species among patches within metacommunities was too frequent to allow competitive exclusion but we have no data to evaluate this possibility. It is important to note that the work by Gross and Cardinale [11] and Cardinale et al. [37], which shows that more species can coexist if resources are provided in a balanced supply, refers to diversity as species richness. But the theory on how resource balance affects richness, and simultaneously how richness affects the efficiency in which resources are converted into new tissue [11], can potentially be extended to considerations of evenness: at imbalanced supply, a few species would dominate at the expense of others, whereas more species are more likely to persist at higher abundances at balanced supply, thereby increasing evenness.

At the metacommunity scale, our results agree with the general pattern of nutrient enrichment resulting in increased biovolume, and increased dominance and lower evenness in freshwater microalgae [38]. Contrary to hypothesis 3, however, some monocultures always had higher biovolume than the mixture at each level of P_sup_. But even though there was no transgressive overyielding [19] we found positive net diversity effects for biovolume at the highest level of P_sup_, and positive net diversity effects for resource use efficiency (Δ_RUE_) across all levels of P_sup_. In other words, there was evidence of non-transgressive overyielding, which indicates that diverse algal communities were more efficient in converting resources into new tissue compared to the average monoculture. Patterns of diversity effects at the local patch scale generally reflected those at the metacommunity scale, with some clear effects of stoichiometry. For example, complementarity effects were significantly positive only at low and intermediate N∶P ratios at P_sup_ V. Resource use efficiency (RUE) was higher than expected only for imbalanced N∶P at the highest level of P_sup_. From these results it is clear that the effects of species richness were highly context dependent, changing with both the total supply of limiting resources and their ratios.

We hypothesized that a positive relationship between evenness and RUE, and thus also evenness and biovolume, would only be apparent at the metacommunity scale because different species would be most efficient under different resource ratios (hypothesis 4). In other words, diversity would be important in a heterogeneous environment because species have different niches, as was observed in the study by Hillebrand and Lehmpfuhl [26]. Indeed, diversity effects on ecosystem processes increases with heterogeneity in real ecosystems [39], and a laboratory experiment has shown that bacterial diversity affects productivity in heterogeneous metacommunities [40]. Furthermore, heterogeneity in the physical environment can also be important [41]. In our study, however, taking into account the effects of P_sup_ and N∶P, realized algal evenness at the metacommunity scale was not positively correlated with RUE. At first glance, our results seem to match those of many previous laboratory experiments with algae, which revealed strong effects of low diversity (single species) on production of biomass (e.g. [42]–[44]). All those previous efforts, however, used local patches with a homogenous resource base. Experiments that explicitly studied the effect of resource heterogeneity on ecosystem processes have yielded mixed results. In an experiment with microalgae [45], the authors contrasted homogeneous and heterogeneous environments by creating sets of patches with either only one N∶P ratio (16∶1) or a range of N∶P ratios (from 4∶1 to 64∶1). Contrary to the authors' hypothesis, one algal species dominated both types of environments, resulting in a positive “selection” effect (see [33]). In contrast, our results suggest that the algal species facilitated each other or were complementary in their use of resources, but that this effect on biovolume was counteracted by some species in monoculture being less productive in species mixtures (Fig 4A; Fig. S2 C). A study with bacteria found that both resource heterogeneity and species richness positively affected metabolic activity [46], but only in few combinations of resource heterogeneity and species richness did species mixtures outperform the best monoculture. This suggests that complementarity was uncommon, probably because species did not greatly differ in niche dimensions, or that negative interactions (e.g. competition) between species was strong. For a guild of fungi, effects of species richness also increased with resource heterogeneity, although patterns were somewhat idiosyncratic [47]. However, it has also been shown that species complementarity effects can be stronger in less heterogeneous environments, indicating that facilitative interactions may sometimes be more important than resource partitioning for positive effects of species richness [48]. In our experiment, even though the five algal species likely have different resource requirements [28], resource heterogeneity and trait diversity were not large enough to overwhelm the effects of a general and strong competitor, the cyanobacterium *Cylindrospermum*. Taken together, there is hitherto limited experimental evidence that species mixtures are generally more efficient than monocultures in using resources in heterogeneous compared to homogeneous environments. Negative interactions among species may indeed be stronger than positive interactions or complementarity, or we have yet to experimentally map resource diversity with the corresponding trait diversity among species. A match between resource heterogeneity and species' resource requirements is key for diversity effects to be apparent [49].

While our results are in line with some other microcosm studies that manipulated resource diversity and resource use, they contrast with the findings of both large-scale positive relationships between freshwater algal diversity and phosphorus use efficiency in Scandinavian lakes and the Baltic Sea [50], and small-scale positive relationships between freshwater microalgae and metacommunity biovolume in the controlled laboratory experiment with heterogeneously distributed resources [26]. As discussed above, a possible explanation for a lack of such patterns in our study is that dispersal among patches was too high, or that other factors affecting species coexistence, such as allelopathy, were important. Further studies examining the importance of resource imbalance and scale are deeply needed to enhance our understanding of the reciprocal relationship between biodiversity and resource supply.

## Supporting Information

Figure S1 **Resource use efficiency (RUE) for monocultures and the species mixture.**
(DOC)

Figure S2 **Biovolumes of each species in mixture, expressed both as observed proportions and as deviance from expected based on monocultures.**
(DOC)

## References

[1] Loreau M, Naeem S, Inchausti P, Bengtsson J, Grime JP (2001). Biodiversity and ecosystem functioning: current knowledge and future challenges.. Science.

[2] Mittelbach GG, Steiner CF, Scheiner SM, Gross KL, Reynolds HL (2001). What is the observed relationship between species richness and productivity?. Ecology.

[3] Cardinale BJ, Hillebrand H, Harpole WS, Gross K, Ptacnik R (2009). Separating the influence of resource ‘availability’ from resource ‘imbalance’ on productivity-diversity relationships.. Ecology Letters.

[4] Waide RB, Willig MR, Steiner CF, Mittelbach G, Gough L (1999). The relationship between productivity and species richness.. Annual Review of Ecology and Systematics.

[5] Rosenzweig ML, Abramsky Z, Ricklefs RE, Schluter D (1993). How are diversity and productivity related?. Species diversity in ecological communities.

[6] Abrams PA (1995). Monotonic Or Unimodal Diversity Productivity Gradients - What Does Competition Theory Predict.. Ecology.

[7] Chase JM (2010). Stochastic Community Assembly Causes Higher Biodiversity in More Productive Environments.. Science.

[8] Currie DJ (1991). Energy and Large-Scale Patterns of Animal- and Plant-Species Richness.. American Naturalist.

[9] Naeem S (2002). Disentangling the impacts of diversity on ecosystem functioning in combatorial experiments.. Ecology.

[10] Kaiser J (2000). Rift over biodiversity divides ecologists.. Science.

[11] Gross K, Cardinale BJ (2007). Does Species Richness Drive Community Production or Vice Versa? Reconciling Historical and Contemporary Paradigms in Competitive Communities.. American Naturalist.

[12] Naeem S, Bunker DE, Hector A, Loreau M, Perrings CH (2009). Biodiversity, ecosystem functioning, & human wellbeing, an ecological and economic perspective..

[13] Schmid B, Balvanera P, Cardinale BJ, Godbold JA, Pfisterer AB, Naeem S, Bunker DE, Hector A, Loreau M, Perrings CH (2009). Consequences of species loss for ecosystem functioning: meta-analyses of data from biodiversity experiments.. Biodiversity, ecosystem functioning, and human wellbeing: an ecological and economic perspective.

[14] Cardinale BJ, Matulich KL, Hooper DU, Byrnes JE, Duffy E (2011). The functional role of producer diversity in ecosystems.. American Journal of Botany.

[15] Braakhekke WG, Hooftman DAP (1999). The resource balance hypothesis of plant species diversity in grassland.. Journal Of Vegetation Science.

[16] Tilman D (1982). Resource competition and community structure..

[17] Titman D (1976). Ecological competition between algae: experimental confirmation of resourcebased competition theory.. Science.

[18] Gause GF (1934). The struggle for existence..

[19] Trenbath BR (1974). Biomass productivity of mixtures.. Advances in Agronomy.

[20] Salles JF, Poly F, Schmid B, Le Roux X (2009). Community niche predicts the functioning of denitrifying bacterial assemblages.. Ecology.

[21] Kolasa J, Picket STA (1991). Ecological heterogeneity..

[22] Kane TC, Poulson TL (1976). Foraging by Cave Beetles: Spatial and Temporal Heterogeneity of Prey.. Ecology.

[23] McQuaid CD, Dower KM (1990). Enhancement Of Habitat Heterogeneity And Species Richness On Rocky Shores Inundated By Sand.. Oecologia.

[24] Sterner RW, Elser JJ (2002). Ecological stoichiometry: the biology of elements from molecules to the biosphere..

[25] Interlandi SJ, Kilham SS (2001). Limiting resources and the regulation of diversity in phytoplankton communities.. Ecology.

[26] Hillebrand H, Lehmpfuhl V (2011). Resource stoichiometry and consumers control the biodiversity- productivity relationship in pelagic metacommunities..

[27] Guillard RRL, Smith WL, Chanley MH (1975). Culture of phytoplankton for feeding marine invertebrates.. Culture of Marine Invertebrate Animals.

[28] Quigg A, Finkel ZV, Irwin AJ, Rosenthal Y, Ho TY (2003). The evolutionary inheritance of elemental stoichiometry in marine phytoplankton.. Nature.

[29] Guildford SJ, Hecky RE (2000). Total nitrogen, total phosphorus, and nutrient limitation in lakes and oceans: Is there a common relationship?. Limnology and Oceanography.

[30] Redfield AC (1958). The biological control of chemical factors in the environment.. American Scientist.

[31] Hillebrand H, Dürselen C-D, Kirschtel D, Pollingher U, Zohary T (1999). Biovolume calculation for pelagic and benthic microalgae.. Journal of Phycology.

[32] Magurran AE (1988). Ecological diversity and its measurements..

[33] Loreau M, Hector A (2001). Partitioning selection and complementarity in biodiversity experiments.. Nature.

[34] Matthiessen B, Ptacnik R, Hillebrand H (2010). Diversity and community biomass depend on dispersal and disturbance in microalgal communities.. Hydrobiologia.

[35] Elser JJ, Bracken MES, Cleland EE, Gruner DS, Harpole WS (2007). Global analysis of nitrogen and phosphorus limitation of primary producers in freshwater, marine and terrestrial ecosystems.. Ecology Letters.

[36] Schnitzer SA, Klironomos JN, HilleRisLambers J, Kinkel LL, Reich PB (2011). Soil microbes drive the classic plant diversity–productivity pattern.. Ecology.

[37] Cardinale BJ, Bennett DM, Nelson CE, Gross K (2009). Does productivity drive diversity or vice versa? A test of the multivariate productivity-diversity hypothesis in streams.. Ecology.

[38] Hillebrand H, Gruner DS, Borer ET, Bracken MES, Cleland EE (2007). Consumer versus resource control of producer diversity depends on ecosystem type and producer community structure.. Proceedings Of The National Academy Of Sciences Of The United States Of America.

[39] Tylianakis JM, Rand TA, Kahmen A, Klein A-M, Buchmann N (2008). Resource Heterogeneity Moderates the Biodiversity-Function Relationship in Real World Ecosystems.. PLoS Biology.

[40] Venail PA, MacLean RC, Bouvier T, Brockhurst MA, Hochberg ME (2008). Diversity and productivity peak at intermediate dispersal rate in evolving metacommunities.. Nature.

[41] Griffin JN, Jenkins SR, Gamfeldt L, Jones D, Hawkins SJ (2009). Spatial heterogeneity increases the importance of species richness for an ecosystem process.. Oikos.

[42] Bruno JF, Boyer KE, Duffy JE, Lee SC, Kertesz JS (2005). Effects of macroalgal species identity and richness on primary production in benthic marine communities.. Ecology Letters.

[43] Gamfeldt L, Hillebrand H, Jonsson PR (2005). Species richness changes across two trophic levels simultaneously affect prey and consumer biomass.. Ecology Letters.

[44] Zhang Q-G, Zhang D-Y (2006). Resource availability and biodiversity effects on the productivity, temporal variability and resistance of experimental algal communities.. Oikos.

[45] Weis JJ, Madrigal DS, Cardinale BJ (2008). Effects of Algal Diversity on the Production of Biomass in Homogeneous and Heterogeneous Nutrient Environments: A Microcosm Experiment.. PLoS ONE.

[46] Langenheder S, Bulling MT, Solan M, Prosser JI (2010). Bacterial Biodiversity-Ecosystem Functioning Relations Are Modified by Environmental Complexity.. PLoS one.

[47] Replansky T, Bell G (2009). The relationship between environmental complexity, species diversity and productivity in a natural reconstructed yeast community.. Oikos.

[48] Tiunov AV, Scheu S (2005). Facilitative interactions rather than resource partitioning drive diversity-functioning relationships in laboratory fungal communities.. Ecology Letters.

[49] Ptacnik R, Moorthi SD, Hillebrand H (2010). Hutchinson Reversed, or Why There Need to Be So Many Species..

[50] Ptacnik R, Solimini AG, Andersen T, Tamminen T, Brettum P (2008). Diversity predicts stability and resource use efficiency in natural phytoplankton communities.. Proceedings Of The National Academy Of Sciences Of The United States Of America.

