# Supplementary material for: Effects of Total Resources, Resource Ratios, and Species Richness on Algal Productivity and Evenness at Both Metacommunity and Local Scales
Source: PLoS One. 2011 Jul 6;6(7):e21972. doi: 10.1371/journal.pone.0021972 (PMC3130793; doi:10.1371/journal.pone.0021972)

**Figure S1**

**Fig. S1. Resource use efficiency (RUE) for monocultures and the species mixture.** At the scale of metacommunities (A). At the scale of local patches at N:P 2 (B), N:P 16 (C), and N:P 128 (D). AN = *Ankistrodesmus*, CL = *Chlamydomonas*, CY = *Cylindrospermum*, FR = *Fragilaria*, and GY = *Gymnodinium*. Solid line indicates the mixture.


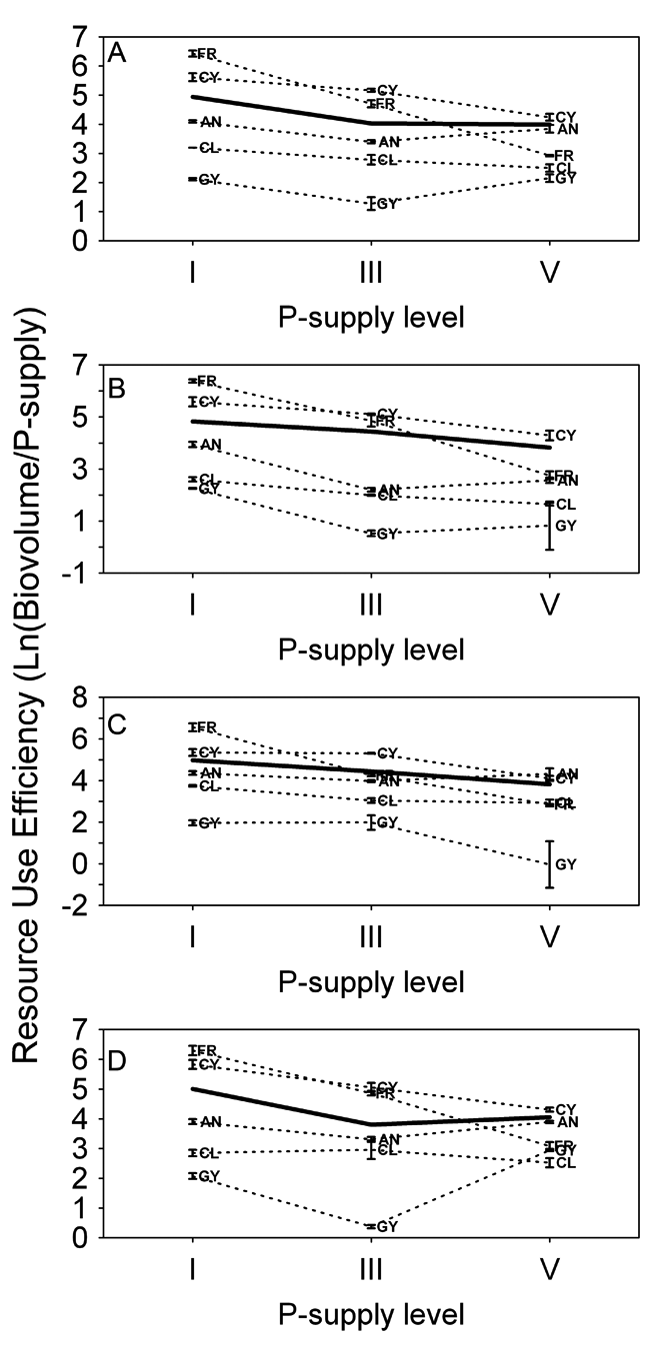

Supplement: Figure S1 — Resource use efficiency (RUE) for monocultures and the species mixture. (DOC) [file pone.0021972.s001.doc]
