# Supplementary material for: Effects of Total Resources, Resource Ratios, and Species Richness on Algal Productivity and Evenness at Both Metacommunity and Local Scales
Source: PLoS One. 2011 Jul 6;6(7):e21972. doi: 10.1371/journal.pone.0021972 (PMC3130793; doi:10.1371/journal.pone.0021972)
Supplement: Figure S2 — Biovolumes of each species in mixture, expressed both as observed proportions and as deviance from expected based on monocultures. (DOC) [file pone.0021972.s002.doc]

**Figure S2**

**Fig. S2. Biovolumes of each species in mixture, expressed both as observed proportions and as deviance from expected based on monocultures.** Proportional biovolumes at the metacommunity scale, in mixture (A) and monocultures (B) for P_tot_ levels I, III, and V. (C) The observed biovolume for each species in mixture minus their expected biovolume in monoculture (10^6^ µm³ mL^-1^).

All calculations for the figure in Appendix S2 were performed on metacommunity-scale data only. For the species mixture we calculated the proportional biovolume of each species as follows:

$$proportion_{i,j}=\frac{biovol_{i,j}}{\sum_{i}^{5} biovol_{i,j}}$$

where *i* is the *i^th^* number of species in metacommunity *j* (3 metacommunities for each P_tot_ level). Proportional biovolumes in the mixture are shown in Fig. S2 A. We did the same for the five monocultures collectively, by dividing the biovolume of species *i* in monoculture in each metacommunity *j(i)* by the sum of all species in monoculture in metacommunities *j(i)*:

$$proportion_{i,j(i)}=\frac{biovol_{i,j(i)}}{\sum_{i}^{5} biovol_{i,j(i)}}$$

The proportional biovolume of species in monocultures is a measure of the expected proportion in mixture in the absence of interspecific competition. Expected proportional biovolumes in the monocultures are shown in Fig. S2 B.

We also calculated for each species the observed biovolume in mixture minus their expected biovolume in monoculture (delta algal biovolume), shown in Fig. S2 C. Expected biovolume was defined as monoculture biomass multiplied by 0.2 (because there were 5 species).
